# Biochemical Engineering Approaches for Increasing Viability and Functionality of Probiotic Bacteria

**DOI:** 10.3390/ijms17060867

**Published:** 2016-06-02

**Authors:** Huu-Thanh Nguyen, Dieu-Hien Truong, Sonagnon Kouhoundé, Sokny Ly, Hary Razafindralambo, Frank Delvigne

**Affiliations:** 1 Natural Products and Industrial Biochemistry Research Group (NPIB), Faculty of Applied Sciences, Ton Duc Thang University, 19 Nguyen Huu Tho, Tan Phong Ward, District 7, 700000 Ho Chi Minh City, Vietnam; 2 Microbial Processes and Interactions (MiPI), Agro-biochem Department, Gembloux Agro-Bio Tech, University of Liège, Passage des Déportés 2, 5030 Gembloux, Belgium; kouhoundes@hotmail.fr (S.K.); soknyly@gmail.com (S.L.); 3 Faculty of Applied Sciences, Ton Duc Thang University, 19 Nguyen Huu Tho, Tan Phong Ward, District 7, 700000 Ho Chi Minh City, Vietnam; truongthidieuhien@tdt.edu.vn; 4 Food technology and Formulation, Agro-Biochem Department, Gembloux Agro-Bio Tech, University of Liège, Passage des Déportés 2, 5030 Gembloux, Belgium; hr436028@scarlet.be

**Keywords:** biochemical engineering, prebiotics, probiotics, exopolysaccharide, sub-lethal stress, survival, cellular robustness

## Abstract

The literature presents a growing body of evidence demonstrating the positive effect of probiotics on health. Probiotic consumption levels are rising quickly in the world despite the fluctuation of their viability and functionality. Technological methods aiming at improving probiotic characteristics are thus highly wanted. However, microbial metabolic engineering toolbox is not available for this kind of application. On the other hand, basic microbiology teaches us that bacteria are able to exhibit adaptation to external stresses. It is known that adequately applied sub-lethal stress, *i.e.*, controlled in amplitude and frequency at a given stage of the culture, is able to enhance microbial robustness. This property could be potentially used to improve the viability of probiotic bacteria, but some technical challenges still need to be overcome before any industrial implementation. This review paper investigates the different technical tools that can be used in order to define the proper condition for improving viability of probiotic bacteria and their implementation at the industrial scale. Based on the example of *Bifidobacterium bifidum*, potentialities for simultaneously improving viability, but also functionality of probiotics will be described.

## 1. Introduction

Probiotics are living microorganisms that are able to promote the health of the host due to their metabolic activities and substances they produce when they are living in the host gastrointestinal tract. This has led to the expansion of their application in many sectors. Probiotics are not only used in food processing [1] but also in medical [2,3], agriculture, and aquaculture industries [4]. Therefore, the global demand of probiotics was valued at $27.9 billion in 2011, and is estimated to reach $44.9 billion in 2018 with 7% annual growth [5]. The consumption of probiotics is increasing despite the fact that probiotics have shown large fluctuations in quality [6], and more specifically loss of viability during downstream processing operations (such as centrifugation and drying) [7], and the fact that they are particularly sensitive to changes in the environment [8]. Classical metabolic engineering toolbox cannot be used in order to improve the phenotypic properties of probiotics, since the use of genetic modification is a limiter for such application [9] One possible alternative is the use of evolutionary engineering. Indeed, it has been previously shown that laboratory evolution in serial batch or continuous culture can potentially lead to improved phenotypes, e.g., with *Lactococcus lactis* cells exhibiting an accelerated acidification rate [10]. However, even if the analysis of the evolved phenotypes can be sped up by modern high-throughput sequencing tools, this approach is difficult to apply to the adaptation of strains to downstream processing operations [11]. Currently, new approaches based on biochemical engineering can potentially lead to high yield of living cells in large scale production and high survival rate during storage [7]. Most of the studies performed in this area have been primarily focused on improving the robustness and viability of cells during downstream processing operations (*i.e.*, mainly freeze-drying and to a lesser extent spray-drying) [8,12,13,14]. However, under these conditions, microorganisms are exposed to fast and intensive stresses. On the other hand, upstream conditions present more potentialities for the application of a diversity of stresses on cells exhibiting different physiological behavior [15]. Indeed, during a cultivation operation, the growing microbial population displays a whole range of phenotypic traits depending on the initial cell density, growth conditions, growth rate, growth phase, *etc.* In this context, two important features have been reported, *i.e.*, cells in stationary phase are more robust than in exponential phase and cells previously exposed to stress in a given range become more resistant to this kind of stress (or a stress involving genes located into the same regulon, *i.e.*, involving the pleiotropic effect of stress response pathways) [16]. However, in order to successfully apply such strategies, a series of important questions have to be addressed:

### What Type of Stress to Apply?

- How to adjust the level of stress, *i.e.*, the amplitude, but also the frequency at which the environmental stimulus is applied to the culture?
- How to implement the results at the industrial level?

These questions will be specifically discussed in the next sections of the paper. More generally, this paper will be focused on the description of the non-genetically modified organisms (non-GMOs) strategies used for producing high-quality probiotic bacteria as well as the technologies used for increasing the robustness of cells during fermentation, downstream processing and storage, for improving the functionality of probiotic in the gastrointestinal tract. As an example, the cultivation of *Bifidobacterium bifidum* will be more thoroughly described, as well as the technological parameters and the resulting physiological features leading to an improved probiotic quality [8,13].

Since these responses will occur at a phenotypic level only, manufacturers will have to implement these sub-lethal stresses upon generation of each probiotic batch. Manufacturers will also have to ensure that costs incurred implementing these stresses are covered by subsequent downstream bacterial survival.

## 2. Exploiting Stresses for Improving Microbial Trait: A Non-GMOs, Biochemical Engineering Approach

### 2.1. Mechanistic View of Stress Response of a Microbial Population

Bacteria, in general, are able to face a diversity of environmental conditions using complex transcriptional networks. Indeed, most of the responses are controlled at the transcriptional level in bacteria due to their defense mechanisms to improve survival in harsh environments. The response of probiotics depends on differential expression of the associated genes, this expression being governed by the nature, the amplitude, and the frequency of the applied stress. Generally, probiotics responds to environmental stresses by activating different regulons, including: (1) implication in energy metabolism operations; (2) transcription and translation associated operations; (3) implication in nucleotide metabolism and amino acid biosynthesis operations; and (4) cell envelope and cell wall-associated operations. These operations produced chaperone proteins that support the folding of misfolded proteins, proteases that hydrolyze irreversibly damaged proteins, transport systems to stabilize osmolality, catalases and superoxide dismutase to decompose reactive oxygen species, as well as proton pumps, decarboxylases enzymes, transporter factors to control intracellular pH [17,18], and exopolysaccharides that protect the cells during drying process [8,13]. Moreover, these proteins are also involved in the increase of the adhesion capacity, in S-layer production, and in fatty acid metabolism of probiotic bacteria [19].

Stress robustness is a desirable trait for the production of starter cultures. In the case of probiotics, this robustness must be accompanied with traits favoring the development of the microbial population in the gut [7]. As stated before, stress resistance is greater during stationary phase or with cells growing at a lower growth rate, and with cells having encountered prior exposure to stress [15,20]. However, there are two additional important features to be considered in order to fully understand the mechanisms behind microbial stress response. The first one is that, upon exposure to stress (*i.e.*, when population shifts from an environmental condition A to B), microbial cells belonging to the same population can react differently. This phenomenon has to be attributed to the phenotypic plasticity of microbial cells and stochasticity resulting from the low amount of biochemical species involved in stress response pathways [21]. Phenotypic heterogeneity must be considered when cell robustness is targeted since this mechanism is also implied in stress resistance and fitness in response to environmental challenge. This mechanism where a given population exhibits different subpopulation with different growth and stress resistance characteristics is called bet-hedging [22]. Thus, care has to be taken for the selection of the appropriate phase of the culture where the majority of the cell among the population is in an appropriate phenotypic state. 

These two important features can be captured by the use of modern experimental approach such as flow cytometry. Flow cytometry is a single cell technique giving access to cell individuality and that can release, when coupled to appropriate tagging, to a fast determination of cell physiology. Parameters that can be determined by flow cytometry are ranging from global ones, *i.e.*, viability and vitality [23], to more specific ones, *i.e.*, activation of specific genes, co-factor analysis, and accumulation of intracellular metabolites [24]. Additionally, this technique can now be directly coupled to cultivation devices by using appropriate interface, allowing the profiling of microbial physiology at a single cell resolution during the whole culture [25,26]. This last feature is particularly interesting for selecting a specific cultivation phase where stresses have to be applied for the induction of microbial robustness [21,27]. The second important feature of microbial response to stress is the multiplicity of pathways that can be potentially implied in the response. Indeed, microbial cells have evolved by optimizing their fitness to fluctuating environments. System biology approaches (or in other words, “omics” based technologies) have thus to be considered for controlling stress response during bioprocesses [28]. Cultivation and analytical technologies related to the monitoring of stress response in process-related conditions will be considered in the next section. 

### 2.2. Scale-Up/Down: How to Cope with Industrial Constraints?

**Figure 1:**
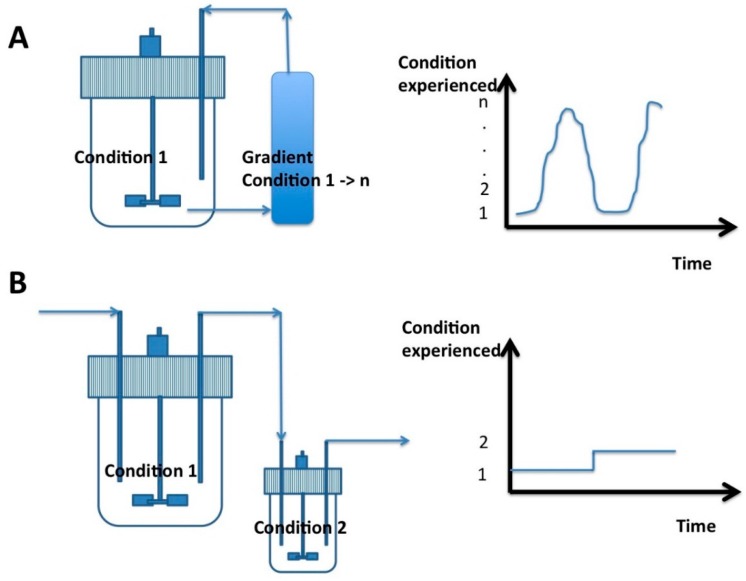
Fermentation devices that can be used for improving the viability of probiotics: (**A**) Batch cultivation devices based on the recirculation of microbial cells between a stirred bioreactor and a plug-flow recycle loop. By his way, gradient of environmental conditions can be generated at the level of the recycle loop allowing to expose the cells to progressive and recurrent stresses [8]; (**B**) Continuous cultivation devices based on two stirred bioreactors in series. In this case, only two distinct cultivation conditions can be generated. However, this device had the advantage of maintaining the microbial population at a given stage by chemostat control [7]. Typical single cell traces (*i.e.*, the history of environmental conditions met by cells) are represented for each bioreactor. As shown, the plug-flow recycle loop allows for the generation of much more variable environmental states from 1 to n (depending on the operating conditions).

The idea by which cultivation conditions can be modulated for increasing the viability and the functionality of probiotics is not new [7,14]. However, additional efforts have still to be provided for the identification of specific stimuli that can be efficiently implemented in industrial fermentation. For example, continuous cultures of probiotic strains can be performed in two-compartment reactors [29,30]. The first reactor is conducted under normal conditions, whereas the second reactor placed in series is operated under stress conditions. By this way, several stresses can be performed on the same culture. However, this approach does not take into account the diversity of amplitude and frequency that can be experienced by a microbial culture in industrial bioreactors. An alternative strategy relies on the use of another cultivation device, also based on two compartments, but with a continuous recirculation of the cells between the two compartments. Such devices have been previously used as a scale-down system in order to reproduce environmental fluctuations expected in large-scale bioreactors, where the lack of mixing efficiency leads to the appearance of concentration gradients [31]. A typical system comprises a stirred bioreactor connected to a plug-flow recirculation loop, this last section being used for generating a specific gradient field (typically, gradient of pH, nutrient, dissolved oxygen or carbon dioxide and temperature are considered). By this way, it is possible to generate a diversity of stress conditions resulting from the superimposition of the flow path followed by a cell through the gradient field [32]. Previous studies have shown that, if this system generally leads to lower biomass yield coefficient, cells obtained at the end of the process exhibit an increased robustness [33,34]. This increase of robustness has to be attributed to the continuous exposure of cells to fluctuating environmental conditions, impairing growth performance but stimulating various stress response pathways [35]. Such two-compartment scale-down reactor have been previously used in order to increase the robustness of *Bifidobacterium bifidum* by applying various temperature stress [8]. The comparison of fermentation devices that can be used for improving the viability of probiotics shown in Figure 1.

All these cultivation devices can be used for the generation of specific environmental conditions leading to improved cell robustness. However, in order to be able to appropriately drive stress resistance during bioprocesses, appropriate monitoring methods are needed. For this purpose, omics-based technologies (*i.e.*, microarray, RNASeq, *etc.*) can be applied in order to explore the fitness landscape of the strain (*i.e.*, by comparing stress conditions to normal culture conditions) [10,28]. On this basis, appropriate biosensors can be used as dynamic parameter for the estimation of stress robustness during cultivation and conditioning. Such biosensor has been developed for the detection of *Bacillus* species robustness on the basis of the generation of ROS [16]. However, probiotic quality does not rely only on robustness, but also to their functionality in the gut. We will show in section 4 that choosing appropriate upstream conditions can possibly link cell robustness and functionality.

## 3. Biotic and Abiotic Factors Yielding to an Increase of Probiotics Quality

### 3.1. Environmental Stresses

It has been previously documented that environmental stress improved the viability of probiotics after downstream processing [8,36,37,38]. Sub-lethal treatments drive the physical or chemical stress factors that do not kill cells or organisms but force them to adapt for survival. Many studies suggest that a wide range of environmental stresses were responsible for causing injuries and death to cells [18]. However, the sub-lethal treatment caused by environmental factors (high and low temperature, pH, osmotic, peroxide, bile salt, redox potential, starvation, *etc.*) allows the bacteria to activate their defense system against intracellular damage, and enhances the viability of probiotics after drying and storage [17,39].

#### 3.1.1. Exposure to Sub-Lethal Heat Stress

Excessive environmental temperatures cause protein denaturation, nucleic acid injury, membrane damage [40], and DNA damage in bacteria [41]. However, when bacteria are exposed to harsh temperatures, they reprogram their metabolic systems for responding to changes in their environmental temperature [36]. 

Sub-lethal temperature stress response has been largely studied in probiotics in recent years [42]. There has been much evidence that the exposure of cells to sub-lethal temperature increases their survival under stressful conditions. The survival rate of *Lactobacillus* species increased 10–1000-fold, depending on the strain, when exponential phase cells were exposed to a sub-lethal temperature before challenging with a lethal temperature [42]. The improved viability rate of *Lactobacillus* species that were subjected to sub-lethal temperatures is shown in Table 1.

The survival rate of *Lactobacilli* increased significantly compared to the control when cells underwent the sub-lethal temperature before exposure to the lethal temperature [38,43]. Additionally, improved viability rates after exposure to sub-lethal temperature were from 5 to 700 folds higher than the control. These results confirm that the sub-lethal temperature stress improves the heat resistance capacity of Lactobacillus species.

#### 3.1.2. Exposure to Sub-Lethal Cold

When the temperature is reduced suddenly, bacteria undergo physiological cell changes, such as changes in membrane fluidity, DNA supercoiling, protein synthesis, and impaired replication of DNA and RNA [42]. To decrease the negative effects of cold temperature, the activity of bacteria must be retained at low temperatures, thus leading to a development of transient adaptive cold shock response in probiotic bacteria. 

Cold tolerance is one of the most important features of probiotic bacteria, because they must be exposed to cold temperatures during their production, storage and preservation (centrifugation, freezing, freeze-drying, and cold storage). Moreover, probiotic bacteria are also used in the food industry, and these products should be frozen before consumption. Bacteria have to maintain their viability and their probiotic properties at low temperature during the processing and preservation.

Freezing causes cell damage, not only by forming water crystals that injure the cell membrane, but also by changing the osmotic pressure gradient of the nutrients inside and outside of cells [44]. There is well-documented evidence that cells that are subjected to low temperature (e.g., 15 °C for 2 h) before freezing, have an increased survival rate: approximately 10-fold for *Lacbacillus sanfranciscensis* CB1, 25-fold for *Lactobacillus plantarum* DB200 and *Lactobacillus brevis* H12, and 100-fold for *Lactobacillus plantarum* 20B [45]. The viability of *Lactobacillus plantarum* L67 increased by 8% when subjected to cold-stress [46]. According to Fang *et al*. [47], *Streptococcus thermophilus* was exposed to cold shock, and its tolerance to a simulated gastric juice and bile solution improved. Cells exposed to low temperature presented higher synthesis of unsaturated and cyclic fatty acids, leading to a higher unsaturated and saturated membranes fatty acid ratio and an increase in the synthesis of four specific proteins leading to cryo-adaptation of probiotics [4].

Besides using the sub-lethal stress, many studies have reported that adaptation stress is also used to improve the survival of probiotic after the drying process, as well as in the production and preservation of cells. The adaptation to low temperature improved the viability of *Lactobacillus plantarum* by 2-log-units (CFU/mL) [48], and *Lactobacillus paracasei* NFBC338 by 5-log-units [36,49], respectively.

Therefore, cold shock improved the gastric resistance capacity (pH 2.5 and 2.8) and the viability of *Streptococcus thermophilus* after 12 h of incubation [50].

#### 3.1.3. Osmotic Stress

Sodium chloride is often used to induce osmotic stress in probiotics; the effect of this osmotic stress has been reported by many studies. When exposed to the osmotic stress adaptation, probiotic viability was a 0.83-log-unit (CFU/g) higher compared to the unstressed cells [51]. Probiotics adapted to NaCl stress were significantly more resistant to heat stress of *Lactobacillus paracasei* NFBC 338 [36,52] and bile [53] than non-adapted control cells. However, the presence of NaCl enhanced the growth of *Lactobacillus plantarum* Lp 813 [48]. On the other hand, *Lactobacillus rhamnosus* HN001 presented significant improvement of their viability in storage at 30 °C after pre-stressing with 0.6 M NaCl [37]. Besides this, trehalose is used for improving the heat tolerance but its effect varies, depending on the strain [53].

#### 3.1.4. pH and Adaptation

Adaptation to low pH allowed the bacteria to improve their resistance to lethal acid conditions [54,55,56]. When subjected to acid stress conditions, the stress tolerance of the *Bifidobacterium longum* and *Bifidobacterium catenulatum* [57] and freeze-drying survival of *Oenococcus* *oeni* cells improved [58]. However, the viability of *Bifidobacterium longum* subsp. longum BBMN68 increased by 70-fold after exposing cells to pH 4.5 for 2 h prior to lethal pH 3.5 for 120 min [59]. Palmfeldt *et al.* [60] observed the highest survival rate after freeze-drying when the cells were grown at pH 5, and harvested after 2.5 h compared to those at pH 6 in the stationary phase (review by [42]). However, less is known about the effect of duration of exposure to the stress stimulus and multi-stresses on viability of probiotic bacteria. In fact, very few data have been reported on improving viability by exposing cells to the stress stimulus during fermentation of probiotic bacteria. The two-stage continuous fermentation process was used for the production of stress-adapted probiotics, which increased tolerance to hydrogen peroxide, simulated gastric and intestinal juices, antibiotics and freeze-drying [29]. Moreover, cell immobilization positively affected the resistance of probiotic bacteria in ways such as increases in cell tolerance to quaternary ammonium sanitizers, hydrogen peroxide, freeze-drying, gastrointestinal conditions, low pH and improving their insoluble exopolysaccharide production (review by [7]). Additionally, the heat shock two-compartment bioreactor, which exposed the cells to sub-lethal temperatures, lead to a significant increase in cell recovery after freeze-drying [8]. Although mono-stress is largely studied on diverse probiotic bacteria, multi-stress pre-adaptation has been less investigated. The viability of multi-stress-adapted probiotics was enhanced under gastrointestinal conditions. The mix of multi-stress-adapted probiotics showed effects against foodborne pathogens [61]. Furthermore, as a practical example, combining sub-lethal heat stress and CO_2_ transfer intensification for optimizing *Bifidobacterium bifidum* survival rate will be investigated

#### 3.1.5. Redox Potential

Probiotics are particularly sensitive to oxygen. There are well-documented studies on their protection against oxygen toxicity during the fermentation process. Strategies to avoid oxygen toxicity include use of special aerobic or oxygen consuming strains [62], use of oxygen scavengers (e.g., ascorbate, l-cysteine, and tea extract) [63,64], microencapsulation [65], adaptation to oxidative stress [66], or use of adequate packaging material [67]. Deoxygenation by inert gases (*i.e.*, nitrogen, carbon dioxide and hydrogen) cause a decrease in redox potential and an improvement survival ability of *Bifidobacterium* *bifidum* after drying and while in storage [66,68,69,70]. It is noted that carbon dioxide can not only maintain the anaerobic growth conditions of *Bifidobacterium longum* JBL05 but also enhance the cell concentrations and the exopolysaccharide (EPS) secretion [68], and activate the colony formation of *Bifidobacterium* species [71]

#### 3.1.6. Carbon Dioxide

Carbon dioxide has been used for deoxygenizing the culture media before cultivating the probiotic bacteria [13,69]. Some evidence demonstrated that the carbon dioxide was able to regulate, not only the physiology, but also the energy metabolism, as demonstrated by upregulation of enzymes involved in glycolysis and homolactic fermentation of *streptococcus thermophiles* [72]. That led to the improvement of the viability of *Bifidobacteria longum* and Bifidobacteria [68,71]. However, carbon dioxide can, not only maintain the anaerobic growth conditions of *Bifidobacterium longum* JBL05, but also enhance the cell concentrations and exopolysaccharide (EPS) secretion [68]. Additionally, there are many reports on the role of phosphoenolpyruvate carboxylase (PpC) and carbamoyl-phosphate synthase on probiotic, which catalyzed the fixation of CO_2_ to aspartate, arginine, and uracil biosynthesis, respectively [72,73,74,75,76,77]. In short, the CO_2_ was used both for creating the anaerobic conditions that improved the probiotic viability and for biosynthesizing amino acids.

### 3.2. Co-Cultivation Strategies

Previous studies have shown that co-culturing different probiotic strains can lead to probiotic mix with improved properties. For example, the viability of probiotic *Bacillus* species. was improved by co-culturing with *Streptococcus thermophilus* and *Lactobacillus bulgaricus* rather than as a mono-culture [78]. Additionally, ruptured *Streptococcus thermophilus* cells released intracellular components after the early exponential growth phase, which enhanced the growth and function of *Lactobacillus casei* in co-culture [79]. Moreover, when aerobic and anaerobic facultative microorganisms were used in co-culture, the oxygen uptake metabolism was improved; the content of O_2_ in the media was decreased, leading to a decrease of oxygen toxicity for probiotic bacteria. The viability of *Lactobacillus paracasei* H9 had increased gastrointestinal tolerance due to co-cultured yeast [80]. Similar results were reported on the co-culture of *Bifidobacterium bifidum* with *Lactobacillus* species and *Streptococcus* species, as the probiotic cell concentration remained constant during their storage [81].

However, this strategy is difficult to implement to large-scale conditions, considering the difficulties associated with the synchronization of growth of multiple strains in the same bioreactor.

### 3.3. Other Factors

Cryo-protectants such as maltodextrin and glycerol were added to probiotic biomass to support cells against adverse environments during drying (review by [82]). Cells were also protected by microencapsulation using alginate calcium before the drying process. Antioxidants, inert gases, and high-oxygen consuming strains were used for reducing the oxygen content of culture or fermentation media [83].

## 4. Increasing Functionalities of Probiotics by Exploiting Stress Responses of Probiotic Bacteria

### 4.1. Production of Functional Macromolecules

**Figure 2:**
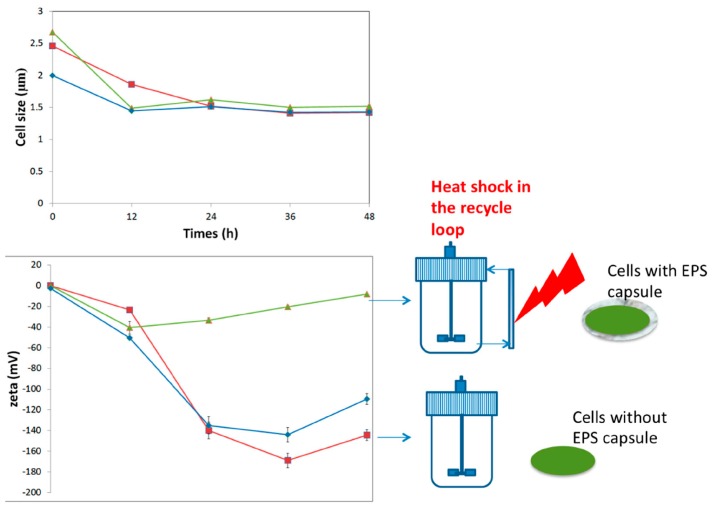
Effect of sub-lethal heat shock (37–42 °C when cells are crossing the recycle loop, two-compartment reactor, see Figure 1) on cell size and zeta potential. Annulation of zeta potential is observed in the case of the bioreactor performed with heat shock. In this case, stress leads to the excretion of an EPS capsule that can be exploited in order to further increase the robustness of the strain during downstream processing operations (adapted from Nguyen [8]). (square: reference, well-mixed bioreactor, diamond: cold-shock two-compartment bioreactor, triangle: heat-shock two-compartment bioreactor).

Bacteria have developed multiple stress response pathways, organized in network, giving them the potentiality to adapt to environmental stress. The responses to environmental stress of bacteria have been the subject of many studies [18,41,44,45,84]. In addition, the main strategies against stress conditions are the synthesis of internal molecules (*i.e.*, functional proteins), the modification of the fatty acid composition of the cell membrane and the alteration of cell surface by inducing synthesis of exopolysaccharides (e.g., EPS forming an extracellular capsule in the case of *Bifidobacteria*). Indeed, following exposure to environmental stress, many macromolecules can be involved. A proteomics analysis of the biological response of probiotics under stress showed an increase of functional proteins such as G*roEL*, *ClpL1*, *Hsp family*, *DnaK*, *GrpE*, *DnaJ*, *ClpS*, ATPase family, *Csp*A, NADH oxidase and NADH peroxidase. These are heat and cold shock proteins, or members of the ATP and enzyme family [49,85,86]. Moreover, for adaptation to environmental stress, the probiotics can also alter their cell surface by producing extracellular exopolysaccharides, changing their fatty acids composition, and changing surface associated proteins (oligosaccharides building protein) [87,88,89]. These physiological mechanisms, and more particularly those related to excretion of EPS, can be exploited during upstream operations in order to increase the robustness of the microbial cells (Figure 2).

### 4.2. Exopolysaccharide Production

The production of EPS by Lactic acid bacteria and *Bifidobacteria* has been subjected to large studies [90,91]. Most of which reported the effect of medium composition and growth conditions such as carbohydrates, temperature and pH of growth medium, and stress condition factors [92] to EPS production. In general, there are two types of EPSs; the first type is bound to cell wall (also called attached EPS, or capsular polysaccharides), whereas the other is unattached to cell wall and moves easily in culture (slime EPS, or free EPS) [93,94]. Most EPS production by lactic acid bacteria (LAB) are heteropolysaccharide [95]. EPS biosynthesis was a complex biochemical process with the participation of severe extracellular enzymes, which were secreted and localized to the cell wall of bacteria. After synthesis, EPS attaches to the cell wall and carries out its biological functions as a capsule, or is released into the medium as slime [94,96,97].

EPS biosynthesis involves four phases, which are described below.

#### Phase 1: Transportation of monosaccharides and disaccharides from the environment into the cell

In the cell, sugars are degraded to d-glucose and α-d-galactose. Two sugars are broken down by the glycolytic pathway or phosphoketolase pathway [92,96,98]. For EPS biosynthesis, the d-glucose and α-d-galactose will be converted into the form of sugar-1 Phosphate [97]

#### Phase 2: Synthetic sugar-1 Phosphate

Most sugar-6-phosphates are hydrolyzed by the glycolytic pathway for energy and secondary metabolite substances. Under some stress conditions, the sugar-6-phosphates are transformed into sugar-1-phosphates, which are important precursors for the synthesis of EPS repeating units [97]. 

#### Phase 3: Synthesis of sugar nucleotides or the EPS repeating uni

The EPS repeating units, such as the UDP-glucose, UDP-galactose, dTDP-rhamnose, UDP-GlcNAc, GDP Fucose, dTDP-Rhamnose, and UDP-Galactofuranose, are synthesized from sugar-1 phosphates. This process is done by a sequence of enzymes encoded by *galU*, *galE*, *rfbA*, *rfbB*, *rfbC*, and *rfbD* genes. [97,99]. The EPS biosynthesis process of *Lactobacillus rhamnosus* strains is controlled by a gene cluster distributed on a DNA region of 18.5 kb encoding the 17 open reading frames (ORFs) and five promoters, of which one is involved in rhamnose biosynthesis [100]. Twenty-one genes (e.g., *epsCBAKLDEFGHIJMNOPQRSTU*) were predicted to participate in the EPS synthesis process but the participating genes varied for each strain. The size of the EPS specific gene cluster coding for EPS production of *Streptococcus thermophilus* Sfi 6 was 14.5 kb, it contains 13 genes, and is located on the chromosome, whereas in *Lactobacillus lactis* NIZO B40, the size is 12 kb, it contains 14 genes, and is located on the plasmid [101]. Contrary to LAB, the reports of genetic analyses of EPS biosynthesis in members of the genus *Bifidobacterium* are very scarce in literature. The EPS cluster of *Bifidobacterium animalis* subsp. lactis IPLA-R1 (previously named A1dOxR) was limited to a DNA region of 54.3 kb, including 42 predicted genes flanked by a transposase-encoding gene. The genes in this EPS cluster were similar to those detected in LAB-EPS clusters when compared by homology studies [102]. The EPS cluster of *Bifidobacterium breve* UCC20003 consists of chromosomal DNA regions of 25.6 kb with 20 predicted genes, which are located in two adjacent regions (eight genes in the *eps*1 operon and 10 genes in the *eps*2 operon) [103].

#### Phase 4: EPS polymerization and export to the surrounding medium

**Figure 3:**
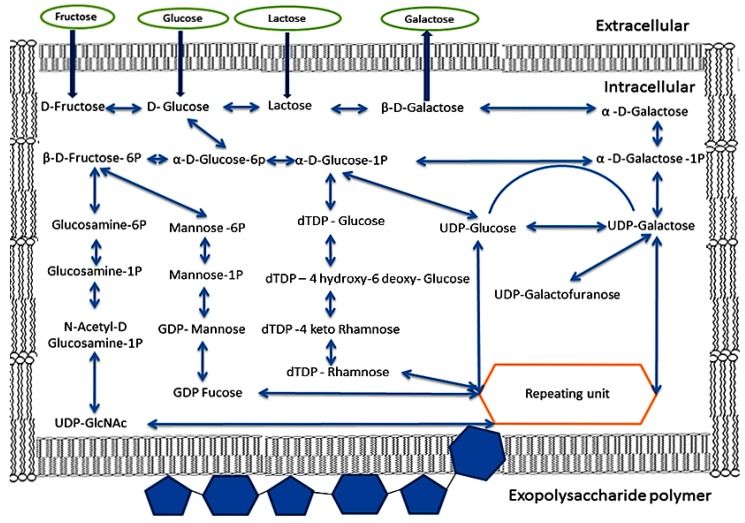
Diagram showing the biosynthetic pathways involved for the production of exopolysaccharides from lactose, fructose, galactose and glucose by lactic acid bacteria.

The assembly of the EPS repeating unit in the cell was done by priming-GTF. This enzyme transfers an EPS repeating unit to the lipophilic carrier molecule (C55-polyprenyl phosphate) located in the cytoplasm membrane of cell [104]. This transfer triggers the addition of a repeating unit to the hetero-EPS molecule [92,99]. When an EPS repeating unit is built into the EPS polymer chain, an export process begins to move the finished unit to the extracellular face of the cytoplasmic membrane, and to attach it to the cell wall or surrounding medium [51]. The EPS gene cluster contains 14.52 kb of DNA encoding 13 genes (*epsA* to *epsM*) in of *Streptococcus thermophilus* Sfi6 and 12 kb of DNA region encoding 14 genes in *Lactobacillus lactis* subsp. Cremoris NIZO B40 [101]. The structure of the EPS gene cluster showed homology with glycosyl transferases specifically required for the biosynthesis of the EPS-repeating unit at central regions of genes. The homologous enzymes participated in polymerization and export of EPS repeating units (chain-length control, export, and polymerization) that were discovered in two regions flanking the central region, and a region of regulation placed at the beginning of the gene cluster [92]. The biosynthesis of EPS from monosaccharides and disaccharides is summarized in Figure 3.

### 4.3. Role of Probiotic Exopolysaccharide

Exopolysaccharides (EPS) are carbohydrate polymers that are produced by a vast variety of microorganisms as a capsule covering their cell surface. From many years, the application of EPS has been an interesting research topic. EPS is used in diverse sectors, such as food, feed, biotechnology, cosmetics, and medicine. However, the main roles of EPS are described below.

### 4.4. Cell Protection Properties

Some studies indicated that EPS play important roles in bacteria in response to environmental stress, and more specifically, during the drying process [105]. It has been shown that an EPS layer is formed at the cell surface upon water removal. This EPS layer has a protective effect, which is in accordance with the increased viability observed for the microbial cells after drying [106]. One of most important role of EPS is less damage and protecting the cell against the harsh conditions of environment (e.g., protection against desiccation, antibiotics, toxic compounds and osmotic stress [107]). There is strong evidence that the amount of EPS produced is related to the thermoresistance and stimulation of the viability of probiotics during freezing and freeze-drying [91], as well as during storage of freeze-dried cells [105,108,109,110,111,112,113,114,115]. The addition of EPS to the food matrix can help probiotics in enhancing the microbial adhesion to the intestinal epithelium and, as a result, the gut colonization is improved by beneficial microorganisms. A steady colonization of the gut is a desirable feature of probiotics [50]. In addition, it is known that EPS excretion leads to an improved robustness of the strains in front of the harsh conditions that can be found in the human gastric system. It also improved the functionality of probiotic strains, e.g., by inhibiting the development of pathogens in the host [111]. Nguyen *et al.* reported that EPS improved the viability of *Bifidobacterium bifidum* after freeze-drying [13].

### 4.5. Prebiotic Properties

In previous studies, the EPS produced from the probiotic are heteropolysaccharides that are formed by linking monosaccharides in their structure (e.g., rhamnose, glucose, galactose, *etc*.) [92]. Indeed, the EPS composition was similar in prebiotics (*i.e.*, the polysaccharide inulin or fructo-oligosaccharides (FOS) derived from various crops or from sucrose) and galacto-oligosaccharides (GOS). Many reports show that the EPS produced from LAB have prebiotic characters such as non-digestibility, fermentable by the intestinal microbiota, and selective stimulation of growth and activity of intestinal bacteria [116,117]. EPS produced by *Lactococcus lactis* subsp. cremoris are readily degraded through the digestive passage in rat and humans [91], while EPS from *Lactobacillus lactis* ssp. cremoris B40, *Lactobacillus sakei* 0–1, *Streptococcus thermophilus* SFi20, *Lactobacillus helveticus* Lh59, and *Streptococcus thermophilus* SFi 39 and SFi 12 are readily degraded by both soil and fecal inocula [118]. Excreted levan-type EPS from *Lactobacillus sanfranciscensis* showed a bifidogenic effect, although it increased the growth of *clostridia* [97]. The size of *Bifidobacteria* population increased significantly in fecal samples when co-fermented with EPS producing strain *Pediococcus damnosus* in human [119]. There have been many reports that the exopolysaccharides produced by *Lactobacillus* species have been linked to potential health benefits [90].

## 5. Application of Probiotic Functionalities

### 5.1. Food Applications

Lactic acid bacteria have been used in fermentation of foods such as dairy, fermented vegetables, and fermented meats. Their main roles are food safety and preservation, the improvement of food texture, the production of aroma and flavor for food, the production of functional food and changing the food’s nutrition due to their metabolic substances, such as chemical food additives (*i.e.*, nitrite, sulfite, propionic acid, sorbic acid, benzoic acid, bacteriocin, and exopolysaccharide). Those substances are applied in food preservation technology [120]. However, the consumer demand for foods with health benefits is rising quickly and that has led the food industry to develop new products such as functional foods containing probiotic bacteria in dairy products and fermented food [121]. There is a rapidly growing demand for incorporating probiotics into food but there is a significant problem with fluctuating viability of probiotics during processing. Industries need a new probiotic that remains stable during processing, storage, handling, transportation and subsequent use in functional foods [7,122]. In food applications, exopolysaccharides play functional roles such as improving the texture of food, prebiotics, and food additives [106]. The synbiotic, combining strong probiotics with prebiotics, will respond to industrial demand. 

### 5.2. Pharmaceutical Applications

The application of biochemical bioengineering for the production of probiotics was not only for improving the viability but also to produce EPS with prebiotic characteristics, when probiotics and prebiotics combine to form the synbiotic [13,123]. The term synbiotic is used for describing products that are composed of both probiotics and prebiotics [124]. Synbiotics are used in the functional food industry in ways such as the addition of synbiotics to infant formula. There is well-documented evidence that probiotics and prebiotics protect against colon cancer, aid gut flora by consuming the resistant starch, and induce the chemopreventive enzyme glutathione transferase π in the colon of the rat. Together, these decrease the load of genotoxic agents and increase production of agents that deactivate toxic components in the gut. Synthesized butyrate can also lower cancer risk in the colon [125]. Meanwhile, probiotics play the important role of correcting abnormalities of colonic flora induced by stress [3]. Probiotics show capacities for antitumor, antiulcer, immunomodulatory, antiviral, and cholesterol lowering activities [92,107]. Furthermore, some strains of Bifidobacteria (*i.e.*, *Bifidobacterium breve* YIT4014 and 4043, and *Bifidobacterium bifidum* YIT4007) and their EPS also presented antiulcer activity in rats. *Bifidobacterium bifidum* BF—1 is capable of gastroprotection, as it reduces acute gastric damage by enhancing the production of gastric mucin in a rat model and alleviating gastric symptoms in humans [126]. EPS production from some probiotic bacteria with these characteristics have been expected to correlate with their capacity to regulate the immune response of the host, eliciting both the innate response and adaptation [116,118,127]. In addition, EPS also improves the adherence of bacteria and colonization of human gut [103,128].

## 6. Conclusions

Exposing cells to appropriate environmental stress potentially lead to an increase of probiotic viability. Additionally, probiotic bacteria respond to environmental stresses by producing specific substances, such as exopolysaccharides and proteins. These substances protect the cells from downstream processing operations. EPS could also play a relevant role in cross talk between the EPS-producing bacteria and the gut environment of the host, promoting by this way health beneficial effects. EPS is applied largely in sectors such as the functional food, dairy, medical, and biomaterial industries, but this review shows extended potentialities for using adapted biochemical engineering approaches for the production of synbiotics (a combination of probiotics and prebiotics) allowing the combination in a cost-effective and industrially implementable way of probiotics robustness and functionality. 

## Figures and Tables

**Table 1:** Effect of sub-lethal temperature conditions on survival and heat tolerance to lethal temperature in *Lactobacillus* (*Lb.*) strains during the exponential growth phase review by De Angelis [42].

Strain	Lethal Condition	Surviving Cells (%)	Sub-Lethal Condition	Improved Viability Rate
*Lb. helveticus* LH212	63 °C, 20 min	0.1–1	52 °C, 20 min	11
*Lb. acidophilus* NCFM	63 °C, 20 min	0.1–1	50 °C, 20 min	27
*Lb. acidophilus* LA1–1	60 °C, 30 min	0.003	53 °C, 30 min	166
*Lb. casei* LC301	54 °C, 20 min	0.1–1	42 °C, 20 min	5
*Lb. paracasei* NFBC338	60 °C, 10 min	ND	52 °C, 15 min	300–700
*Lb. collinoides*	52 °C, 30 min	0.48	42 °C, 90 min	24

The viability rate is calculated as the ratio: survival of adapted cells (%)/survival of control cells (%). The controls correspond to non-adapted cells to sub-lethal condition before exposure to lethal condition. ND: Not determined.

## Abbreviations

EPS	exopolysaccharides
DNA	Deoxyribonucleic acid
RNA	Ribonucleic acid
NADH	Nicotinamide adenine dinucleotide
ATP	Adenosine triphosphate
UDP	uridine 5′-diphosphate
GDP	guanosine 5′-diphosphate
TDP	Thymidine diphosphate
LAB	Lactic acid bacteria
